# Effects of minipuberty disruption on the expression of sexual behavior in female mice

**DOI:** 10.1038/s41598-024-82653-8

**Published:** 2024-12-28

**Authors:** Thomas Torres, Caroline Parmentier, Céline J. Guigon, Sakina Mhaouty-Kodja, Lydie Naulé

**Affiliations:** 1 Sorbonne Université, CNRS UMR8246, INSERM U1130, Neuroscience Paris Seine – Institut de Biologie Paris Seine, Paris, France; 2 Université Paris-Cité, CNRS, Inserm, Biologie Fonctionnelle et Adaptative, Paris, F-75013 France; 3 3PHM, Pathophysiology and Pharmacotoxicology of the Human Placenta, pre & post-natal Microbiota, Université Paris-Cité, Inserm, Paris, F-75006 France

**Keywords:** Minipuberty, Females, Sexual differentiation, Reproductive behaviors, sex steroids, Sexual dimorphism, Infertility, Endocrinology

## Abstract

Sex steroids influence early organization of neural structures involved in expression of sexual behavior. A critical perinatal period during which testosterone surges occur has been identified in male rodents. Data are lacking for females, whose ovarian activity starts later in the postnatal period. Minipuberty has been described in female as a first postnatal period of gonadotropic axis activation. We transiently disrupted the minipubertal period, using daily injections of GnRH antagonist or aromatase inhibitor. The results showed unchanged puberty initiation and mild disturbance of estrus cyclicity. Minor modifications of some components of sexual behavior (olfactory preference, ability to attract male) were observed, but had no impact on lordosis behavior. Interestingly, letrozole-treated female mice showed increased anxiety state level at adulthood. This period therefore does not seem to be a key organizational window for female sexual behavior but appears to participate in the organization of neural structures involved in anxiety-related behavior.

**Supplementary Information:**

The online version contains supplementary material available at 10.1038/s41598-024-82653-8.

## Introduction

Sexual reproduction relies on optimal display of sexually dimorphic behaviors expressed by sexual partners. These include in rodents (i) the emission and sensing of olfactory and auditory cues, which inform on the hormonal and receptive state and attract the partner, and (ii) the postures adopted afterward to ensure insemination, such as lordosis behavior for females, mounts, thrusts and intromissions for males^1–3^.

Sex steroid hormones play a key role in the early organization of the neural structures involved in the expression of male and female behaviors. These processes have been extensively studied for half a century in males. However, the precise developmental period during which sex steroids act in females still needs to be documented. In rodents, it is indeed well established, since the seminal work of Phoenix et al.^4^, that testosterone released during the perinatal period by fetal and neonatal testes and its neural metabolite, estradiol, induce a permanent organization of the neural circuitry controlling male sexual behavior^5^. This consists in a potentiation of male (masculinization) and an inhibition of female (defeminization) behavioral characteristics, resulting in the expression of male-typical behaviors and the inability to adopt the female posture (lordosis).

In female rodents, there was a long-standing view that feminization of the neural circuitries regulating fertility and reproductive behaviors might occur as a default pathway. This idea stems from the fact that rodent ovaries do not produce sex steroids during the fetal and perinatal periods^6^. At these stages, the female brain is also protected from the masculinizing effects of maternal (and male sibling) estradiol through its selective binding to the α-fetoprotein^7^. In mice, ovaries start releasing estradiol around postnatal day (PND) 7 and then rise to reach a maximum at puberty. The prepubertal increase of estradiol has been shown to participate in brain feminization^8,9^. For instance, constitutive deletion of *cyp19a1*gene, encoding the aromatase that metabolizes testosterone into estradiol, has been shown to reduce lordosis behavior in adult mutant female mice^8^. In these mice, estradiol supplementation from PND15 to PND25 partially restored the behavioral deficiency, while earlier administration of estradiol between PND5 and PND15 did not^9^. These studies suggested that the period lasting from PND15 to PND25 may represent one of the key postnatal periods involved in the feminization of the neural circuitry underlying female sexual behavior. However, these *cyp19a1* knockout mice are depleted of estrogens from the embryonic period. They also present high levels of testosterone, which could directly or indirectly interfere with neural processes in the female brain.

More recently, high levels of estradiol were detected in the serum of female mice between PND12 and PND17, resulting from a massive and transient gonadotropin surge^10^. This minipubertal hypothalamic-pituitary-gonadal (HPG) activity may regulate uterus and mammary gland development but its role in female reproductive function remains unknown^11^.

The present study aims to determine the precise role of the minipubertal period in the organization of the neural circuits controlling female reproductive function and sexual behavior. For this purpose, we used two pharmacological models to transiently disrupt the ovarian production of estrogens during minipuberty. In the GonadoSTOP model, C57BL6/J female mice were injected from PND10 to PND16 with a GnRH antagonist (Ganirelix^®^) to alter the gonadotropic axis. Such treatment was shown to suppress gonadotropin release and reduce estradiol levels by half after only two days of treatment^10,12^. In the second model, C57BL/6J female mice were treated from PND10 to PND17 with letrozole an inhibitor of aromatase, the enzyme that metabolizes testosterone into estradiol. As aromatase blockade increases the levels of testosterone, we added two other groups treated with letrozole and flutamide (an androgen receptor antagonist) or with flutamide alone. For both models, we monitored body weight, assessed puberty initiation (age at vaginal opening and first estrus) and analyzed estrous cyclicity in adulthood. Afterwards, female mice were ovariectomized and primed with estradiol and progesterone to measure their sexual behavior under normalized hormonal levels. We performed tests for olfactory preference, ability to attract the male partner and lordosis behavior. In addition, general behaviors, including locomotor activity and anxiety-related behavior, was assessed.

## Results

### Effects of minipubertal treatment with Ganirelix^®^ (Experiment 1)

#### Pubertal onset, estrous cyclicity and other physiological parameters

**Fig. 1:**
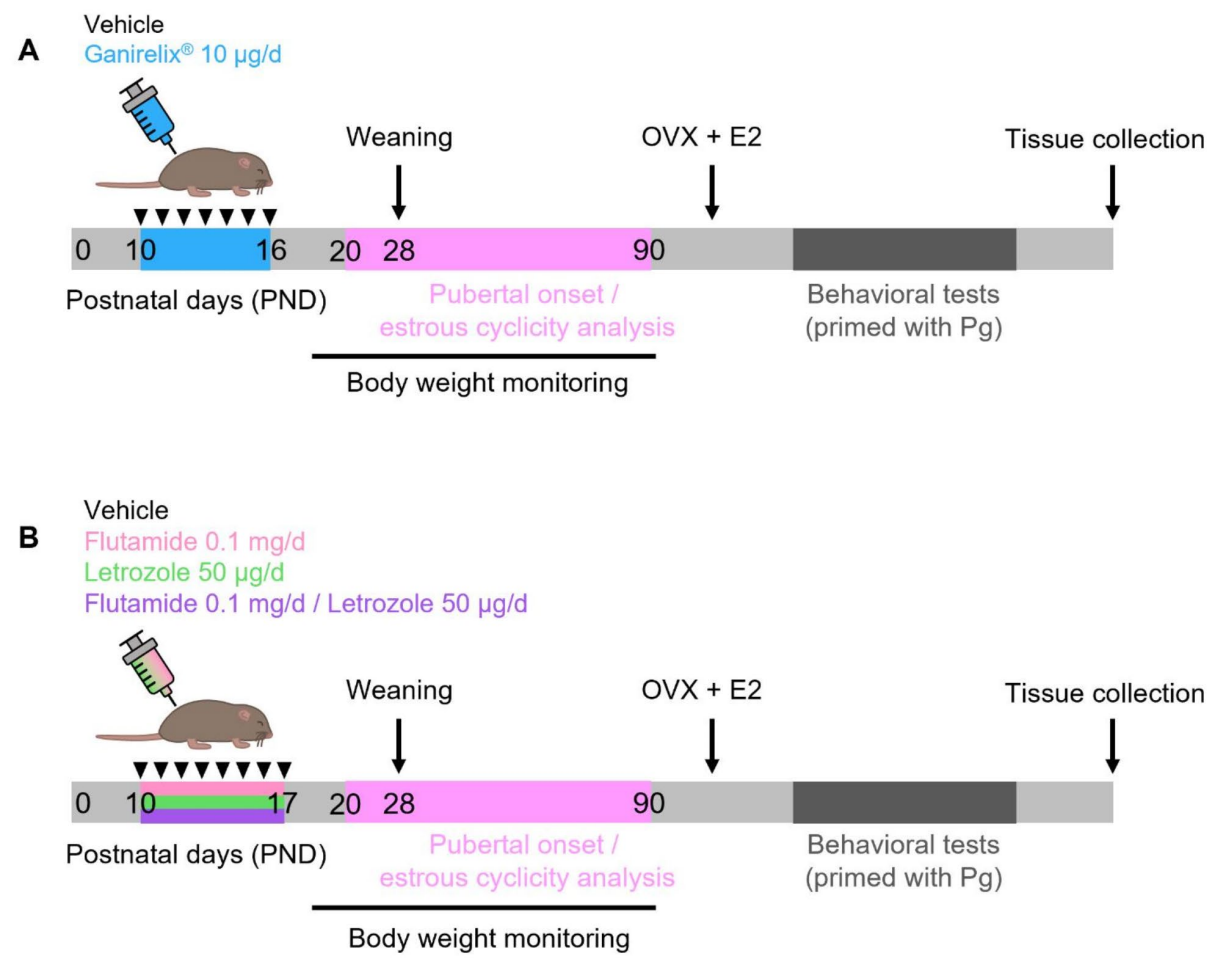
Schematic representation of the experimental design.** (A)** In Experiment 1, female mice were daily treated with the vehicle or Ganirelix^®^ at the indicated doses from postnatal day 10 (PND10) to PND16. **(B)** In Experiment 2, female mice were daily treated with the vehicle, flutamide (F), letrozole (L) or both (F/L) at the indicated doses from PND10 to PND17. For both experiments, body weight, pubertal onset and estrous cyclicity were monitored in intact females. Females were then ovariectomized and received estradiol implants (OVX + E2) and were primed with progesterone (Pg) before each behavioral test. At the end of behavioral tests, females were euthanized and tissues were collected.

**Fig. 2:**
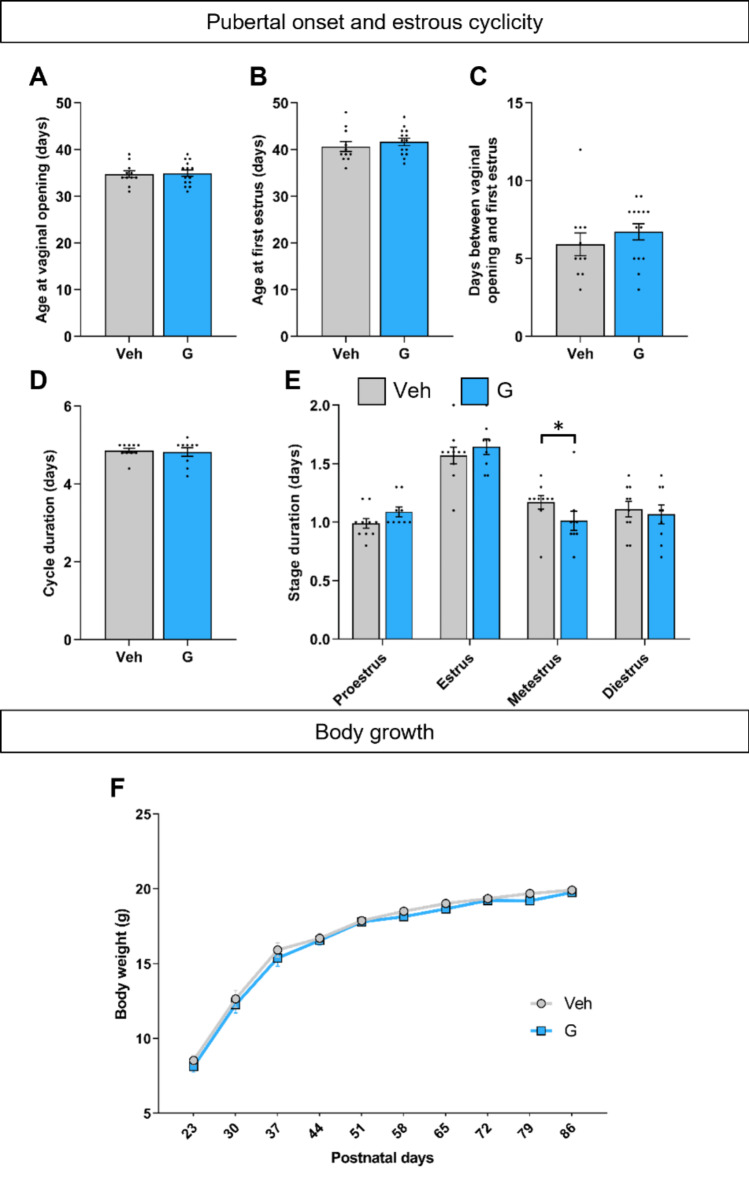
Effects of minipubertal treatment with Ganirelix on pubertal onset, estrous cyclicity and body growth.** A-C.** Female mice treated with the vehicle (Veh) or Ganirelix (G) from postnatal (PND)10 to PND16 were analyzed for the age at vaginal opening (**A**) and first estrus (**B**) as well as the interval between these two pubertal markers (**C**). **D-E**. The estrous cycle was monitored in adult controls and Ganirelix-treated females. The duration of the estrous cycle (**D**) and of each stage (**E**) are indicated; (*p < 0.05 for the metestrus phase). **F**. Body weight was followed every week from PND20 to PND86 for each female. Data are presented as means ± S.E.M. (*n* = 9–15 per treatment).

Postnatal C57BL6/J females were injected with vehicle or Ganirelix for 7 days from PND10 to PND16 (Fig. 1A). The ages at vaginal opening and first estrus were monitored for both groups. The results in Fig. 2A-B shows that there were no differences between the controls and Ganirelix-treated females for the age at vaginal opening (*p* = 0.84) and first estrus (*p* = 0.44). The duration between vaginal opening and first estrus was also comparable between the two treated groups (*p* = 0.15; Fig. 2C).

In adult females, we monitored the estrous cycle for 5 weeks. One vehicle and 5 Ganirelix-treated females showed irregular cycles and were not included in the detailed analysis. A Fisher’s exact test indicated no effect of the treatment on this parameter (*p* = 0.18). Further analysis showed that the treatment did not affect the mean cycle duration (*p* = 0.75; Fig. 2D). Detailed analysis of the estrous cycle (Fig. 2E) showed a comparable duration of the estrus and diestrus phases between controls and Ganirelix-treated females (*p* = 0.45, *p* = 0.68; respectively). In contrast, the metestrus phase was shorter by 13.6% in the Ganirelix group (*p* = 0.0228), while a tendency to increase was observed for the proestrus phase (*p* = 0.077).

Females from vehicle- and Ganirelix-treated mice were weekly monitored for their body weight until ovariectomy. This showed no effect of the treatment (F_(1, 26)_ = 0.57, *p* = 0.45), but an effect of age (F_(1.317, 34.25)_ = 668.6, *p* < 0.0001) (Fig. 2F). There also was no effect on body weight at vaginal opening (*p* = 0.68) (Figure S3A).

The ovaries collected from controls and Ganirelix-treated adult females at the time of ovariectomy were weighed. No difference between the two groups was observed (0.024% ± 0.002 of body weight in controls versus 0.027% ± 0.001 of body weight in Ganirelix-treated females; *p* = 0.45) (Figure S3B).

#### Behavior

Sexual and general behaviors were assessed in vehicle- and Ganirelix-treated females, which were ovariectomized at the end of estrous cyclicity monitoring and supplemented with estradiol and progesterone.

##### Sexual behavior

**Fig. 3:**
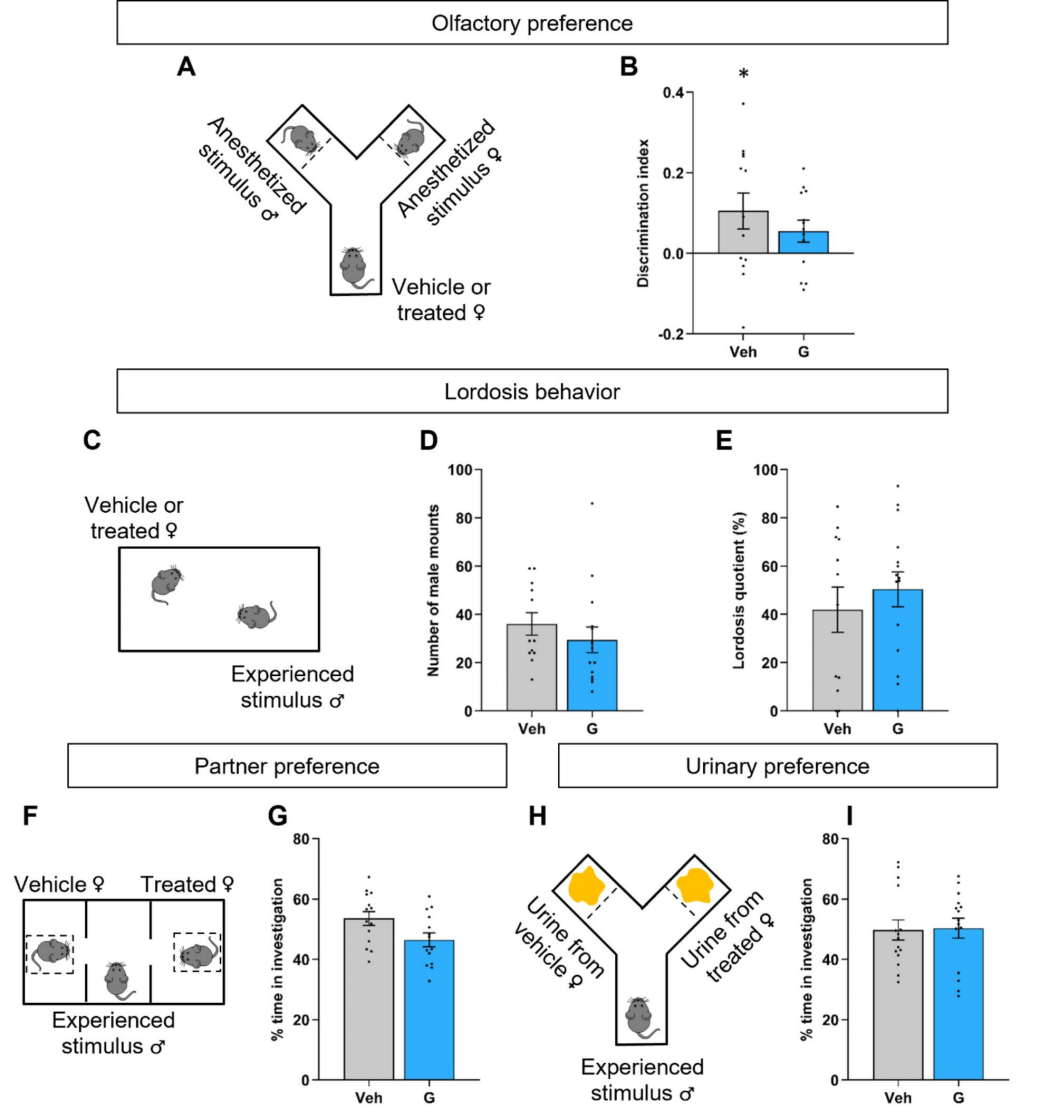
Effects of minipubertal treatment with Ganirelix on female sexual behavior. **A-B.** In the olfactory preference test, female mice treated with the vehicle (Veh) or Ganirelix (G) had choice to chemoinvestigate anesthetized male or female stimuli (**A**). The discrimination index, time spent in male investigation minus the time spent in female investigation divided by the total time of investigation is indicated (**B**). *p < 0.05, positive index for the control group. **C-E**. Lordosis behavior was tested for sexually-experienced females in the presence of sexually experienced males for 30 min (**C**). The number of male mounts (**D**) and lordosis quotient (**E**) are indicated. **F-G**. In the three-chambered test, sexually experienced males had choice to investigate Veh- or G-treated females (**F**). The percentage of time spent investigating each female is indicated (**G**). **H-I**. In the urinary preference test, sexually experienced males had choice to investigate urine from Veh- or G-treated females (**H**). The percentage of time spent investigating each urine stimulus is indicated (**I**). Data are presented as means ± S.E.M. (*n* = 12–15 per treatment).

The ability of females to discriminate between male and female pheromones was tested in a Y-maze with male and female stimuli, both anesthetized and placed at each end of the maze (Fig. 3A). The total time spent in chemoinvestigation was not different between the vehicle- and Ganirelix-treated females (48.35 ± 3.11 s versus 57.66 ± 5.88 s; *p* = 0.1751) (Figure S1A). Two-way ANOVA indicated no effect of stimulus (F_(1, 48)_ = 0.52, *p* = 0.47) and treatment (F_(1, 48)_ = 0.005211, *p* = 0.9428) on the number of entries into the arms (Figure S1B). The calculated discrimination index showed a preference towards male for the vehicle-treated females (*p* = 0.0363; Fig. 3B). Although positive, the discrimination index calculated for Ganirelix-treated females did not reach the statistical significance (*p* = 0.0678). Comparison of the two treated groups did not show differences (*p* = 0.35).

**Fig. 4:**
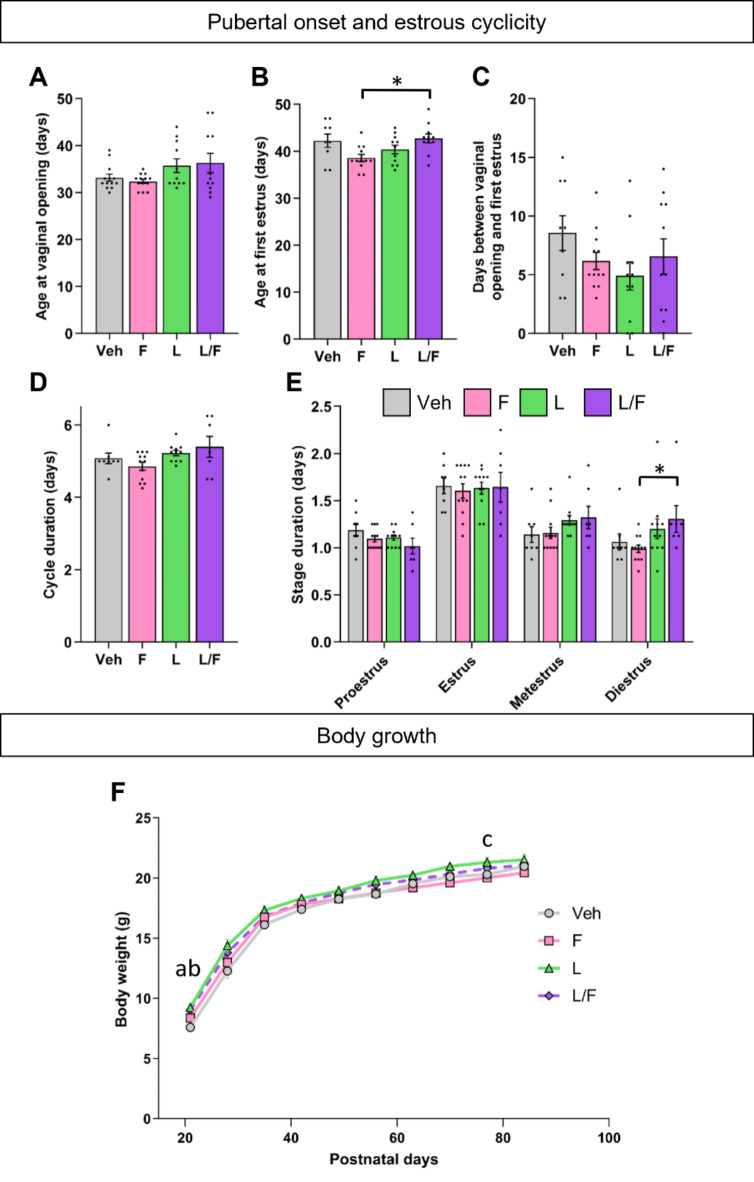
Effects of separate or combined treatment with flutamide and letrozole during minipuberty on pubertal onset, estrous cyclicity and body growth.** A-C.** Female mice treated with the vehicle (Veh), flutamide (F), letrozole (L) or both (F/L) from postnatal day (PND)10 to PND17 were analyzed for the age at vaginal opening (**A**), age at first estrus (**B**) and interval between the two pubertal markers (**C**). *p < 0.05 for the indicated groups. **D-E**. Duration of the estrous cycle (D) and of each stage (E) in adult females. **F**. Body weight was monitored weekly from PND21 to PND86, ^a^*p* <0.05 between Veh and L, ^b^*p* < 0.05 between Veh and F/L, ^c^*p* < 0.05 between F and L). Data are presented as means ± S.E.M. (*n* = 8–13 per treatment).

**Fig. 5:**
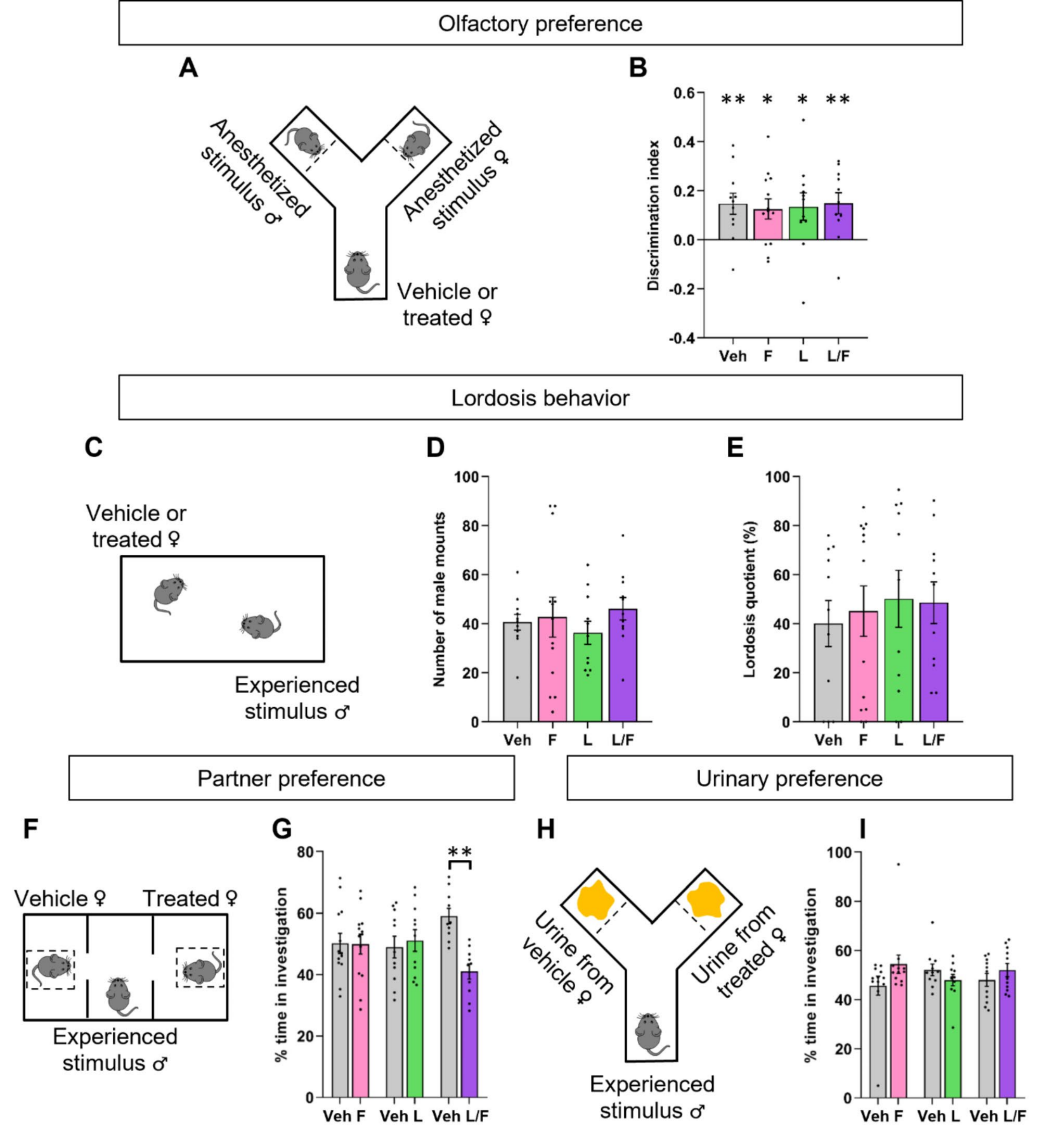
Effects of separate and combined treatment with flutamide and letrozole during minipuberty on female sexual behavior. **A**-**B**. In the olfactory preference test, female mice treated with the vehicle (Veh), flutamide (F), letrozole (L) or both (F/L) had choice to chemoinvestigate anesthetized male or female stimuli (**A**). The discrimination index, time spent by exposed females in male investigation minus the time spent in female investigation divided by the total time of investigation (**B**). *p < 0.05, positive index for the four groups. **C-E**. Lordosis behavior was tested for sexually-experienced females in the presence of sexually experienced males for 30 min (**C**). The number of male mounts (**D**) and lordosis quotient (**E**) are indicated. **F-G**. In the three-chamber test, sexually experienced males had choice to investigate either Veh- or F-, Veh- or L-, or Veh- or F/L-treated females (**p < 0.01 for F/L) (**F**). The percentage of time spent investigating each female is indicated (**G**). **H-I**. In the urinary preference test, sexually experienced males had choice to investigate urine either from Veh- or F-, Veh- or L-, or Veh- or F/L-treated females (**H**). The percentage of time spent investigating each urine stimulus is indicated (**I**). Data are presented as means ± S.E.M. (*n* = 10–13 per treatment).

In mating tests, females were tested in the presence of a sexually experienced male for 30 min (Fig. 3C). Males exhibited a comparable number of mounts in the presence of vehicle- or Ganirelix-treated females (*p* = 0.20) as shown in Fig. 3D. The lordosis quotient did not differ between the two groups of treated females (*p* = 0.47; Fig. 3E).

The ability of vehicle- and Ganirelix-treated females to attract sexually experienced males was assessed using the three-chamber test (Fig. 3F). The total time spent in investigation (*p* = 0.94; Figure S1E), the number of entries into the stimulus compartments (*p* = 0.42; Figure S1F) and the percentage of time spent by males in the investigation of each stimulus (*p* = 0.12; Fig. 3G) were comparable between controls and Ganirelix-treated females.

In the Y-maze test, vehicle- and Ganirelix-treated females were replaced by their urine (Fig. 3H). As for the three-chamber test, the total time spent in stimulus arms (*p* = 0.78; Figure S1I), the number of entries into the two arms (*p* = 0.58; Figure S1J) and the time spent in the investigation of each stimulus (*p* = 0.39; Fig. 3I) were comparable between vehicle- and Ganirelix-treated females.

##### General behaviors

**Fig. 6:**
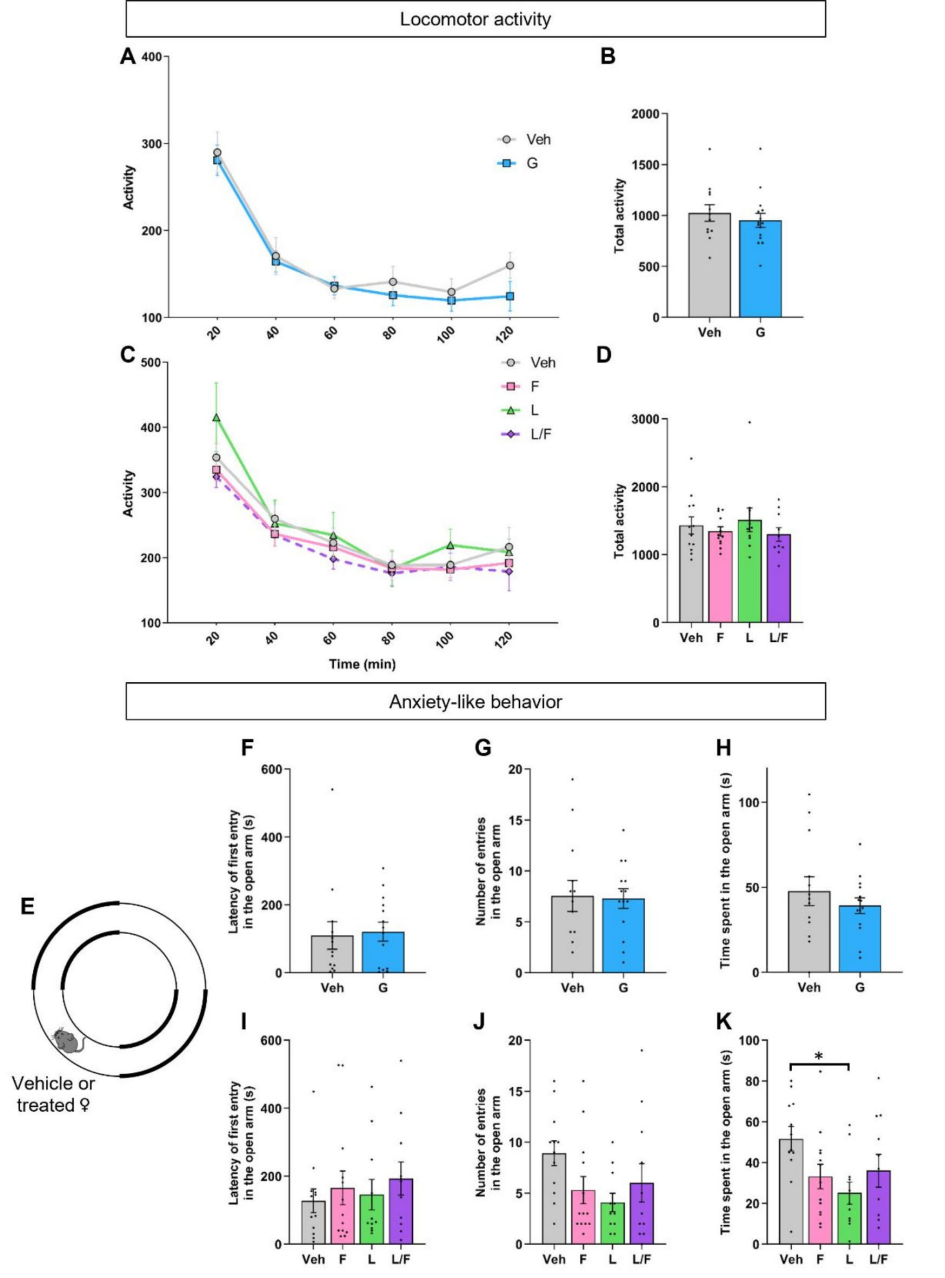
Effect of minipubertal treatment on locomotor activity and anxiety-like behavior. **A-D**. Locomotor activity was monitored in a circular corridor during 2 h. The activity monitored every 20 min (**A**, **C**) or cumulative activity (**B**, **D**) are presented for females treated with the vehicle (Veh) and Ganirelix (G) groups in Experiment 1, and flutamide (F) alone, letrozole alone or both flutamide and letrozole (F/L) in Experiment 2. **E-K**. Anxiety-like behavior was assessed in a 0-maze during 9 min (**E**). The latency to the first entry into the open arm (**F**,** I**), the number of entries into the open arm (**G**,** J**) and the time spent into the open arm (**H**,** K**) are indicated. Data are presented as means ± S.E.M. (*n* = 12–15 per treatment for Experiment 1; *n* = 10–13 per treatment for Experiment 2) *p < 0.05 for the indicated groups.

Locomotor activity of both treated groups was assessed in a circular corridor for 2 h. Two-way ANOVA showed an effect of time (F_(3.337, 83.43)_ = 68.13, *p* < 0.0001) but not of treatment (F_(1, 25)_ = 0.47, *p* = 0.50) (Fig. 6A). Comparison of cumulative activity over the 2 h of the test did not show differences between the two groups (*p* = 0.50) (Fig. 6B).

Anxiety-related behavior was analyzed in an 0-maze paradigm (Fig. 6E). No difference was observed between the two groups in the latency to the first entry into the open arm (*p* = 0.61; Fig. 6F), or the number of entries (*p* = 0.89; Fig. 6G). The time spent in the open arms was also comparable between the treated groups (*p* = 0.38; Fig. 6H).

#### ERα-immunoreactivity

The brains of female mice that were subjected to behavioral tests were analyzed for ERα-immunostaining. It was performed in key hypothalamic regions controlling female sexual behavior, including the activating nucleus VMH and the inhibitory regions, ARC and POA^13,14^. Figure S2 shows a specific nuclear staining in the POA, VMH and ARC. Quantification of the number of ERα-immunoreactive cells showed no difference between vehicle- and Ganirelix-treated females in the POA (*p* = 0.29), VMH (*p* = 0.78) and ARC (*p* = 0.97) (Figure S2A-D).

### Effects of separate and combined treatment with letrozole and flutamide during minipuberty (Experiment 2)

Treatment of female mice with Ganirelix from PND10 to PND16 did not affect the expression of sexual behavior. As Ganirelix treatment only partially suppresses the ovarian production of estradiol^10^, it is possible that the remaining estradiol was sufficient to induce the organization of the neural circuitry underlying female sexual behavior. Therefore, in Experiment 2, C57BL6/J females were daily injected for 8 days from PND10 to PND17 with vehicle (control group), letrozole alone at 50 µg/day to inhibit aromatase activity and thus estradiol production, flutamide alone at 0.1 mg/day, or with both compounds (Fig. 1B). The anti-androgen flutamide was used to counteract the androgenic effects potentially induced by the known elevation of testosterone levels triggered by the blockade of aromatase and thus the metabolization of testosterone into estradiol.

#### Pubertal onset, estrous cyclicity and other physiological parameters

Monitoring of pubertal onset markers showed no effect of the treatment on the age at vaginal opening (*p* = 0.54), while an effect of treatment was observed on the age at first estrus (*p* = 0.018) (Fig. 4A-B). *Post hoc* analysis indicated that females treated with letrozole/flutamide had the first estrus 4.14 days later than those treated with flutamide (*p* = 0.046; Fig. 4B). Analysis of the interval between vaginal opening and first estrus did not show any effect of the treatment (*p* = 0.27; Fig. 4C).

We monitored the estrous cycle for 5 weeks in adult females. One vehicle, one flutamide and 4 letrozole/flutamide-treated females showed irregular cycles and were not included in the detailed analysis. A Fisher’s exact test indicated no effect of the treatment on this parameter (*p* = 0.072). The mean duration of the estrous cycle was unaffected by the treatment (*p* = 0.1317; Fig. 4D). Detailed analysis of each cycle phase duration revealed no effect of the treatment on the duration of the proestrus, estrus and metestrus phases (*p* = 0.29, *p* = 0.99, *p* = 0.06; respectively), although the statistical significance was close to 0.05 for the metestrus phase. In contrast, an effect of treatment was observed for the diestrus phase (*p* = 0.023). Females treated with letrozole/flutamide had a longer duration compared to those treated with flutamide (+ 31.77%; *p* = 0.046) (Fig. 4E).

For body weight monitored between PND20 and PND90, two-way ANOVA showed effects of age (F_(1.652, 66.08)_ = 1545, *p* < 0.0001) and treatment (F_(3, 40)_ = 3.826, *p* = 0.017) (Fig. 4F). *Post hoc* analysis pointed out a transient increase of body weight at PND21 for letrozole-treated females (*p* = 0.015) and letrozole/flutamide-treated groups (*p* = 0.023) compared to the vehicle-treated females, and at PND70 for the letrozole group compared to females treated with flutamide (*p* = 0.027). There also was no effect on body weight at vaginal opening (*p* = 0.095) (Figure S3C).

The weight of ovaries, collected at the time of ovariectomy, was not different between the treated groups (*p* = 0.13) as illustrated in Figure S3D.

#### Behavior

Sexual behavior was analyzed in vehicle-, flutamide-, letrozole- and letrozole/flutamide-treated mice, which were ovariectomized at the end of estrous cyclicity monitoring and hormonally primed as described above.

##### Sexual behavior

In the olfactory preference test (Fig. 5A), there was no effect of the treatment on the total time spent in chemoinvestigation for the four female-treated groups (*p* = 0.1689; Figure S1C). Similarly, no effect of the treatment (F_(3, 86)_ = 0.7207, *p* = 0.5423) and stimulus (F_(1, 86)_ = 0.8275 *p* = 0.3655) was observed for the number of entries into the arms (Figure S1D). All females exhibited preference for the male stimulus (*p* = 0.007, *p* = 0.010, *p* = 0.036, *p* = 0.006, for vehicle-, flutamide-, letrozole- and letrozole/flutamide-treated mice; respectively). One-way ANOVA showed no effect of the treatment on the discrimination index (*p* = 0.98; Fig. 5B).

In the mating test (Fig. 5C), sexually experienced males exhibited comparable number of mounts in the presence of females (*p* = 0.69), regardless of their treatment (Fig. 5D). Furthermore, no effect of the treatment was observed on the lordosis quotient (*p* = 0.72; Fig. 5E).

In the three-chamber paradigm (Fig. 5F), there were no differences in the total time spent by males in the stimulus compartments (*p* = 0.55, *p* = 0.72, *p* = 0.19, for vehicle versus flutamide groups, vehicle versus letrozole groups and vehicle versus letrozole/flutamide groups, respectively; Figure S1G), nor in the number of entries in the compartments (*p* = 0.49, *p* = 0.32 and *p* = 0.31 for vehicle versus flutamide groups, vehicle versus letrozole groups and vehicle versus letrozole/flutamide groups, respectively; Figure S1H). The percentage of time spent by males investigating vehicle- versus flutamide-treated females (*p* = 0.59) and vehicle- versus letrozole-treated females (*p* = 0.8) was not different. In contrast, males spent less time investigating letrozole/flutamide-treated mice than controls (−29.37%, *p* = 0.009; Fig. 5G).

For urinary preference (Fig. 5H), the total time spent in the stimulus arms was not different (*p* = 0.41, *p* = 0.59, *p* = 0.59, for vehicle versus flutamide groups, vehicle versus letrozole groups and vehicle versus letrozole/flutamide groups, respectively; Figure S1K). The number of entries into each arm was also comparable between the three experimental conditions (*p* = 0.45, *p* = 0.83 and *p* = 0.68 for vehicle versus flutamide groups, vehicle versus letrozole groups and vehicle versus letrozole/flutamide groups, respectively; Figure S1L). In addition, the percentage of time spent by males in investigation was not different between the two stimuli for the three experimental conditions (*p* = 0.18, *p* = 0.39, *p* = 0.46 for vehicle versus flutamide groups, vehicle versus letrozole groups and vehicle versus letrozole/flutamide groups, respectively; Fig. 5I).

##### General behaviors

Analysis of locomotor activity by two-way ANOVA showed an effect of time (F_(3.225, 129.0)_ = 87.56, *p* < 0.0001) but not of treatment (F_(3, 40)_ = 0.598, *p* = 0.62) (Fig. 6C). There was no effect of the treatment on the cumulative activity over the 2 h of the test (*p* = 0.76; Fig. 6D).

For anxiety-related behavior (Fig. 6E), there was no effect of the treatment on the latency to the first entry into the open arm (*p* = 0.77, Fig. 6I), but a tendency to decrease was observed for the number of entries although it did not reach the statistical significance (*p* = 0.059, Fig. 6J). One-way ANOVA showed an effect of treatment on the time spent into the open arms (F_(3, 43)_ = 2.995, *p* = 0.041), with reduced time spent in the open arms for letrozole-treated mice compared to vehicle group (−48.51%, *p* = 0.0176) (Fig. 6K).

## Discussion

In this study, using two pharmacological methods, we investigated the impact of minipubertal disruption on later pubertal initiation, estrous cyclicity and female sexual behavior.

Disruption of the gonadotropic axis through GnRH antagonist administration (Ganirelix) during the minipubertal period did not impact the age of puberty initiation, with no change in the age at vaginal opening or first estrus in female mice. Adult monitoring of estrous cyclicity showed reduced duration of the metestrus phase in females treated with Ganirelix, without modification of the total duration of the estrous cycle. It has also been previously shown that minipubertal Ganirelix treatment induces no modification in fertility at 3 months of age, suggesting that the slight treatment effect observed on the duration of the metestrus phase does not appear to impact fertility in adulthood. Surprisingly, 11-month-old treated females showed an increase in the percentage of mice becoming pregnant, suggesting a long-term effect of the treatment on reproductive longevity (Chester et al., preprint available at bioRxiv^15^).

Regarding the treatment with an aromatase inhibitor (letrozole) in the absence or presence of an anti-androgen (flutamide), an effect of treatment was observed on the age at the first estrus. This difference was observed only between the flutamide and letrozole/flutamide groups with no difference compared with vehicles. In adulthood, an effect of treatment was observed on the duration of the diestrus phase of the estrous cycle. Again, difference was noticed between the flutamide and letrozole/flutamide groups. The meaning of such differences in the initiation of puberty and estrous cyclicity between these two groups is not clear. Interestingly, it is documented that androgens through the androgen receptor (AR) play a key role in both follicular development and female neural regulation of the gonadotropic axis^16–19^. It is therefore possible that both treatments affected ovarian function and/or gonadotropic axis differently, leading to the observed differences. Nevertheless, it is important to point out that the age of pubertal onset and the duration of the diestrus phase of the cycle in females treated with letrozole or flutamide alone or in combination were in the range of those observed for the vehicle group. In addition, treatments with letrozole or letrozole/flutamide had slight effects on body weight at PND20, suggesting that these treatments, compared to Ganirelix, transiently affected the somatotropic axis.

At the behavioral level, analyses were performed in ovariectomized and hormonally primed females to test the direct effects of the minipubertal treatments on the neural circuitry underlying sexual behavior. Under such normalized hormonal levels, the discrimination index in the olfactory preference test was slightly affected by the treatment with Ganirelix. However, no significant difference was found with the vehicle group. Olfactory stimulation is mandatory for the activation of the other components of sexual behavior. Lordosis behavior, partner and urinary preferences were not affected by Ganirelix treatment. In agreement with these data, the number of ERα-immunoreactive cells was comparable between controls and Ganirelix-treated females in the neural circuitry underlying sexual behavior (VMH, POA and ARC).

Female mice treated during minipuberty with letrozole, flutamide or letrozole/flutamide showed comparable olfactory preference. In the three-chamber test, females of the letrozole/flutamide group attracted less the males compared to the control group. This effect was not observed in the urinary preference test, suggesting differences in the volatile cues between vehicle- and letrozole/flutamide- treated groups. Despite this reduced partner preference, mating assessed by the lordosis quotient was comparable between treatment groups.

Overall, this comprehensive behavioral analysis shows that under normalized hormonal levels, the expression of female sexual behavior was not or slightly impacted following minipubertal treatment with a GnRH antagonist or aromatase inhibitor, in the absence or presence of flutamide. This confirms a previous study showing that estradiol supplementation between PND5 and PND15 did not restore the expression of sexual behavior in aromatase knockout females^9^. In line with the classic view that neonatal estradiol masculinizes the brain structures involved in sexual behavior, this early postnatal treatment masculinized aromatase knockout females similarly to controls, which both exhibited female-directed (male typical) mounting behavior when treated with testosterone in adulthood^9^. In the same study, estradiol treatment from PND15 to PND25 partially restored the expression of lordosis behavior of aromatase knockout adult females. These and our data strongly support that the period involved in the feminization of the neural circuits underlying female sexual behavior occurs at a later age^9^. Further research will be instrumental to delineate the precise prepubertal/pubertal periods during which neural feminization takes place and determine the underlying mechanisms of action.

In addition to sexual behavior, we assessed general behavior. The results showed no effect of the treatments on locomotor activity. In contrast, an increased anxiety state level was observed in female mice treated during the minipubertal period with letrozole. This effect was likely induced by the transient depletion of estradiol during the minipubertal period, since hormonal levels were normalized in adult females analyzed for this behavior. No such effect was observed with Ganirelix treatment, probably explained by the incomplete reduction in estradiol levels in this model. In previous studies, we have shown that deletion of estrogen receptor (ER) *ERβ* or *ERα *in neural progenitors since embryonic day 10 resulted in increased anxiety-related behavior in adult female mice^20–22^. The present data suggest that estradiol released during the minipubertal period (PND10 to PND17) participates in the organization of the neural structures involved in anxiety-related behavior. It is well known that estradiol in adulthood controls the anxiety state of females. Indeed, elevated level of estradiol during the follicular phase induce an anxiolytic effect^23^. Downstream molecular pathways may imply the serotoninergic system and the expression of tryptophan hydroxylase (TPH) in the dorsal raphe nucleus^24^. For instance, the increase in anxiety-related behavior observed in females deleted for neural *ERβ *was associated with a reduction in TPH2-immunoreactivity in the dorsal raphe nucleus since PND30 (unpublished observation^22^). It remains to be determined whether the estradiol released during the minipubertal period participates in the organization of this neural pathway.

### Perspective and significance

In summary, the evaluation of the impact of minipubertal disruption on puberty initiation, estrous cyclicity and female sexual behavior has shown that estradiol released during this period appears to affect the development of female reproductive function and behavior in subtle ways but is not essential for successful fertility and mating in adulthood. A transient activation of the HPG axis during minipuberty, characterized by high levels of gonadotropin LH (luteinizing hormone) and FSH (follicle-stimulating hormone) and elevated gonadal sex steroid production, occurs also in humans shortly after birth and can last up to 3–4 years in girls^25^. The influence of this period on women fertility remains to be determined. Further studies focusing on later developmental periods (prepubertal/pubertal) will help identify the critical developmental periods for brain feminization of reproductive function. Meanwhile, interestingly, this study revealed that estradiol released during minipuberty may play a role in the organization of anxiety-related behaviors. This is consistent with the well-established role for estradiol in the regulation of women’s anxiety throughout life, whether during the menstrual cycle, menopause or in post-partum anxiety disorders. A better understanding of the critical periods of estradiol action would enable better management of these anxiety troubles.

## Conclusions

In this study, we showed that transient disruption of the minipubertal period, in female mice, induced only minor modifications of estrous cyclicity and some components of sexual behavior (olfactory and partner preferences) without impacting subsequent mating behavior. Interestingly, this study, however, revealed that estradiol released during minipuberty may contribute to the organization of female anxiety-related behavior. Hence, these findings support the view that estradiol acts at different periods of development to organize distinct behaviors. Further research will precise the windows of action and the underlying mechanisms of action.

## Methods

### Animals and treatments

Female mice were obtained from local mating of male and female C57BL/6J mice (Janvier Labs). Animals were maintained at a temperature of 22 °C and subjected to a 12-hour light-dark cycle with the dark phase beginning at 1:30 PM and ending at 1:30 AM. They had *ad libitum* access to both food (A03-10; SAFE) and water.

*Experiment 1*. Female mice were subcutaneously injected each day from PND10 to PND16 with 20 µl of sesame oil alone (control group) or 20 µl of sesame oil containing 10 µg of Ganirelix^®^ (Fyremadel Gé, FERRING), a GnRH antagonist (*n* = 12–15 per treatment, 3 different litters for control- and 4 litters for Ganirelix-treated animals) (Fig. 1A). Under such experimental conditions, Ganirelix treatment reduced estradiol levels by 50% in female mice^10^.

*Experiment 2*. Female mice were subcutaneously injected each day from PND10 to PND17 with 20 µl of sesame oil and ethanol 3% for the vehicle group, or containing either 50 µg of letrozole (a reversible aromatase inhibitor), 0.1 mg of flutamide (a non-steroidal anti-androgen), or both (letrozole/flutamide) (*n* = 10–13 per treatment, 3 different litters for control- and 4 litters for letrozole-, flutamide- and letrozole/flutamide-treated animals) (Fig. 1B). The concentrations of letrozole and flutamide were chosen based on previous studies in mice^26,27^. Administration of 50 µg per day of letrozole reduced estradiol level below the limit of assay sensitivity, as in ovariectomized females^27^. In a prenatally androgenized mouse model, daily injection of the same concentration of flutamide was efficient to block androgen receptor signaling and restore estrous cyclicity and GnRH neuron circuit integrity, while unaffecting ovarian and adrenal gland weights^26^.

For both Experiments 1 and 2, female mice were weaned at PND28 and identified by numbers. No information on the treatment groups were available during the analyses.

### Pubertal onset, estrous cyclicity, body and organ weights

The age at pubertal onset was monitored by daily examination of vaginal opening since PND20 (Fig. 1A-B). Once vaginal opening was observed, vaginal smears, flushed with physiological saline (NaCl 0.9%), were collected daily to determine the age of first estrus. Vaginal smears were collected daily until PND90 to examine estrous cyclicity. Microscopic examination of the vaginal smears, following hematoxylin/eosin staining, allowed identification of the estrous cycle phases. The mean duration of the estrous cycle and the number of days spent in each stage were quantified.

Body weight was assessed from PND21 to PND90. The ovaries were collected and weighed at the time of ovariectomy two weeks before the beginning of behavioral assessment.

### Behavioral tests

Experiments were carried out under red-light illumination, occurring 2 h after lights were turned off. All sessions were recorded via videotaping for subsequent analysis. The testing sequence was proceeded as following: 1st test of lordosis behavior for sexual experience, olfactory preference test, locomotor activity monitoring, 2nd test of lordosis, 0-maze test, three-chamber test for partner preference and Y-maze for urinary preference. Behavioral tests were separated by at least three days of complete rest or habituation to the following test. The stud males used were sexually experienced three times before the beginning of behavioral tests. All devices used in these tests, except for the animal home cages, were cleaned with a solution of 10% ethanol between trials.

#### Preparation of females

Female mice were ovariectomized two weeks before the beginning of behavioral tests under general anesthesia and received a 1 cm subcutaneous SILASTIC™ filled with 50 µg of estradiol benzoate (Sigma-Aldrich) diluted in 30 µL sesame oil and sealed at each end with SILASTIC™ adhesive as previously described^20,28,29^. Five hours before each behavioral test, females were primed with a subcutaneous injection of progesterone (1 mg/100 µL).

#### Olfactory preference

Olfactory preference of female mice was tested in an enclosed plexiglass Y-maze, following a previously established protocol^20,30,31^. The maze comprised 3 equivalent arms (25.8 cm length, 11 cm width, 30.7 cm height). Female mice underwent a familiarization period over two consecutive days (day 1 and 2), during which two empty perforated goal boxes were positioned at each end of two arms for a duration of 10 min. On the testing day (day 3), females were placed in the empty arm and had choice for 10 min between a sexually receptive female and an intact male, both placed within the goal boxes in the two opposing arms. To prevent social interaction, the stimuli were anesthetized. Observations included the time spent in chemo-investigation of the stimuli and the number of entries into each arm of the maze. The discrimination index was calculated as the difference in time spent investigating the male (M) versus the female (F), divided by the total investigation time.

#### Partner preference tests

##### Three-chamber test

Sexually experienced males underwent a familiarization phase with the testing arena, a three-chambered box (22.5 cm height, 40.5 cm width, and 62.4 cm length; 20.5 cm for the two peripheral chambers and 21.4 cm for the central chamber) for 10 min over two consecutive days^30,31^. During this period, two empty perforated goal boxes were placed within the side chambers of the arena. On the test day, each male was introduced into the neutral chamber and allowed to freely explore for 10 min. A vehicle-treated female was put in a goal box, while the opposite one contained a female treated with Ganirelix (Experiment 1), flutamide, letrozole or flutamide/letrozole (Experiment 2). Females were randomly assigned to either the left or right chamber. The number of entries into each compartment and the duration the male spent sniffing each female were analyzed.

##### Y-Maze test (urinary preference)

Sexually experienced males underwent a familiarization period with the maze for 10 min over two consecutive days^30,31^. On the testing day, males had choice between urine of females from the vehicle group and urine from one of the treated groups, Ganirelix (Experiment 1) or flutamide, letrozole or flutamide/letrozole (Experiment 2), both put inside perforated goal boxes placed at each end of the maze. Urine was collected one hour prior to the test. Urine samples from all females within each treatment group were combined in equal volumes, and 10 µL of this mixture was applied onto a piece of filter paper. Observations were made on the time spent by males chemoinvestigating each stimulus and on the number of entries into each arm of the maze.

#### Elevated 0-maze

The elevated 0-maze consisted in a 56 cm diameter circular track (5 cm width), divided into two open and two enclosed (bordered by 17 cm-diameter walls) quadrants, elevated at 65 cm above the ground. Females were placed in one of the enclosed quadrants and allowed to freely explore the maze for 9 min. Anxiety-like behavior was assessed by measuring the latency to the first entry into the open arms, the number of entries and the time spent in the open arms. A mouse was considered to be in an open arm when the four paws have entered.

#### Locomotor activity

The circular corridor used for activity measurement consisted in two concentric cylinders crossed by four diametrically opposed infrared beams^29,30^. Locomotor activity was recorded each time animals interrupted two successive beams, indicating they had traveled a quarter of the circular corridor. The activity monitored every 20 min and cumulative activity over the 120-min test were reported.

### Immunohistochemistry

For experiment 1, three days after the end of behavioral assessment, females were euthanized 4 to 5 h after progesterone injection through intraperitoneal administration of ketamine (100 mg/kg) and xylazine (10 mg/kg). They were intracardiacardially perfused with NaCl (0.9%) followed by 4% paraformaldehyde (PFA) in Phosphate Buffer (PB). The brains were extracted, post-fixed overnight in 4% PB-PFA, then frozen in tissue freezing medium (Leica) and stored at −20 °C. The brains were then sectioned into 30 μm coronal sections using a cryostat (Leica CM 3050 S).

For immunodetection, the sections were incubated in a solution containing 1X Phosphate Buffer Saline (PBS) − 0.3% Triton with 2% normal donkey serum for 1 h to saturate non-specific binding sites. Incubation with the primary antibody anti-ERα (1:400; Santa Cruz Biotechnology; rabbit) was performed in 1X PBS − 0.3% Triton for 3 days at 4 °C. The sections were washed then incubated with chicken secondary anti-rabbit coupled with Alexa Fluor 488 (1:500; Invitrogen) in 1X PBS − 0.3% Triton for 2 h at room temperature in the dark. The sections were washed, mounted on SuperFrost slides, dried in the dark at room temperature, and then cover-slipped with Mowiol.

The slides were scanned using a Nanozoomer Hamamatsu Scanner (Hamamatsu corporation) at high resolution and analyzed using NDP.view software. The regions of interest, VMH, arcuate nucleus (ARC), and preoptic area (POA), were identified using the Paxinos & Franklin Mouse Brain in Stereotaxic Coordinates Third Edition (2007) (plate 46, plate 43 and plate 30 respectively). After delineating equivalent area zones (0.3 mm², 0.16 mm², and 0.4 mm², respectively), ERα-immunoreactive neurons were counted in the left and right hemispheres.

### Statistics

The data are expressed as the mean ± standard error of the mean. A *p*-value < 0.05 was considered significant. Normality of the data was assessed with the Shapiro-Wilks normality test. Unpaired t-test and Mann-Whitney test were used to compare vehicle- and ganirelix- treated mice (Experiment 1) for vaginal opening, estrous cyclicity, ovarian and uterine weights, ERα-immunoreactivity, olfactory preference, lordosis and anxiety-related behaviors, as well as cumulative locomotor activity. One-way ANOVA and Kruskal-Wallis tests were used to assess the same tests in Experiment 2. Paired t-test and Wilcoxon test were used in both experiments to analyze partner preference in three-chamber and urinary preference tests. Two-way ANOVA was used in both experiments to assess the effect of treatment on body weight, number of entries in the stimulus arms for olfactory preference and 2-hour monitoring of locomotor activity.

## Electronic supplementary material

Below is the link to the electronic supplementary material.

Supplementary Material 1

## Data Availability

The authors declare that the data supporting the findings of this study are available within the paper and its supplementary information files.

## References

[1] Beach, F. A. Sexual attractivity, proceptivity, and receptivity in female mammals. *Horm. Behav.* **7**, 105–138 (1976). 819345 10.1016/0018-506x(76)90008-8

[2] Gutierrez-Castellanos, N., Husain, B. F. A., Dias, I. C. & Lima, S. Q. Neural and behavioral plasticity across the female reproductive cycle. *Trends Endocrinol. Metab.* **33**, 769–785 (2022). 36253276 10.1016/j.tem.2022.09.001

[3] Everitt, B. J. Sexual motivation: a neural and behavioural analysis of the mechanisms underlying appetitive and copulatory responses of male rats. *Neurosci. Biobehav Rev.* **14**, 217–232 (1990). 2190121 10.1016/s0149-7634(05)80222-2

[4] Phoenix, C. H., Goy, R. W., Gerall, A. A. & Young, W. C. Organizing action of prenatally administered testosterone propionate on the tissues mediating mating behavior in the female guinea pig. *Endocrinology* **65**, 369–382 (1959). 14432658 10.1210/endo-65-3-369

[5] Morris, J. A., Jordan, C. L. & Breedlove, S. M. Sexual differentiation of the vertebrate nervous system. *Nat. Neurosci.* **7**, 1034–1039 (2004). 15452574 10.1038/nn1325

[6] Lamprecht, S. A., Kohen, F., Ausher, J., Zor, U. & Lindner, H. R. Hormonal stimulation of oestradiol-17 beta release from the rat ovary during early postnatal development. *J. Endocrinol.* **68**, 343–344 (1976). 176297 10.1677/joe.0.0680343

[7] Bakker, J. et al. Alpha-fetoprotein protects the developing female mouse brain from masculinization and defeminization by estrogens. *Nat. Neurosci.* **9**, 220–226 (2006). 16388309 10.1038/nn1624

[8] Bakker, J., Honda, S. I., Harada, N. & Balthazart, J. The Aromatase knock-out mouse provides New evidence that estradiol is required during development in the female for the expression of Sociosexual Behaviors in Adulthood. *J. Neurosci.* **22**, 9104–9112 (2002). 12388618 10.1523/JNEUROSCI.22-20-09104.2002 PMC6757696

[9] Brock, O., Baum, M. J. & Bakker, J. The development of female sexual behavior requires Prepubertal Estradiol. *J. Neurosci.* **31**, 5574–5578 (2011). 21490197 10.1523/JNEUROSCI.0209-11.2011 PMC3085119

[10] François, C. M. et al. A novel action of follicle-stimulating hormone in the ovary promotes estradiol production without inducing excessive follicular growth before puberty. *Sci. Rep.* **7**, 46222 (2017). 28397811 10.1038/srep46222 PMC5387682

[11] Devillers, M. M., Mhaouty-Kodja, S. & Guigon, C. J. Deciphering the roles & Regulation of Estradiol Signaling during female Mini-puberty: insights from mouse models. *Int. J. Mol. Sci.* **23**, 13695 (2022). 36430167 10.3390/ijms232213695 PMC9693133

[12] Devillers, M. M. et al. FSH inhibits AMH to support ovarian estradiol synthesis in infantile mice. *J. Endocrinol.* **240**, 215–228 (2019). 30403655 10.1530/JOE-18-0313

[13] Mhaouty-Kodja, S., Naulé, L. & Capela, D. Sexual behavior: from hormonal regulation to endocrine disruption. *Neuroendocrinology* **107**, 400–416 (2018). 30326485 10.1159/000494558

[14] Torres, T., Adam, N., Mhaouty-Kodja, S. & Naulé, L. Reproductive function and behaviors: an update on the role of neural estrogen receptors alpha and beta. *Front. Endocrinol.* **15**, 1408677 (2024). 10.3389/fendo.2024.1408677 PMC11228153 38978624

[15] Chester, M. et al. Minipuberty regulates reproductive lifespan and ovarian follicular loss in a mouse model with reduced minipubertal gonadotropin levels. 08.20.608775 Preprint at (2024). 10.1101/2024.08.20.608775 (2024).

[16] Shiina, H. et al. Premature ovarian failure in androgen receptor-deficient mice. *Proc. Natl. Acad. Sci.* **103**, 224–229 (2006). 16373508 10.1073/pnas.0506736102 PMC1324980

[17] Sen, A. & Hammes, S. R. Granulosa Cell-Specific Androgen receptors are critical regulators of ovarian development and function. *Mol. Endocrinol.* **24**, 1393–1403 (2010). 20501640 10.1210/me.2010-0006 PMC2903904

[18] Walters, K. A. et al. The role of Central Androgen receptor actions in regulating the Hypothalamic-Pituitary-Ovarian Axis. *Neuroendocrinology* **106**, 389–400 (2018). 29635226 10.1159/000487762

[19] Devillers, M. M. et al. Androgen receptor signaling regulates follicular growth and steroidogenesis in interaction with gonadotropins in the ovary during mini-puberty in mice. *Front. Endocrinol.* **14**, 1130681 (2023). 10.3389/fendo.2023.1130681 PMC10154677 37152943

[20] Naulé, L. et al. Delayed pubertal onset and prepubertal *Kiss1* expression in female mice lacking central *oestrogen receptor beta*. *Hum. Mol. Genet.* **24**, 7326–7338 (2015). 26464488 10.1093/hmg/ddv430

[21] Trouillet, A. C. et al. Deletion of neural estrogen receptor alpha induces sex differential effects on reproductive behavior in mice. *Commun. Biol.* **5**, 383 (2022). 35444217 10.1038/s42003-022-03324-w PMC9021208

[22] Dombret, C. et al. Effects of neural estrogen receptor beta deletion on social and mood-related behaviors and underlying mechanisms in male mice. *Sci. Rep.* **10**, 6242 (2020). 32277160 10.1038/s41598-020-63427-4 PMC7148327

[23] Walf, A. A., Koonce, C., Manley, K. & Frye, C. A. Proestrous compared to diestrous wildtype, but not estrogen receptor beta knockout, mice have better performance in the spontaneous alternation and object recognition tasks and reduced anxiety-like behavior in the elevated plus and mirror maze. *Behav. Brain Res.* **196**, 254–260 (2009). 18926853 10.1016/j.bbr.2008.09.016 PMC2614898

[24] Suzuki, H. et al. Involvement of estrogen receptor β in maintenance of serotonergic neurons of the dorsal raphe. *Mol. Psychiatry*. **18**, 674–680 (2013). 22665260 10.1038/mp.2012.62

[25] Becker, M., Hesse, V. & Minipuberty Why does it happen? *Horm. Res. Paediatr.* **93**, 76–84 (2020). 32599600 10.1159/000508329

[26] Silva, M. S. B., Prescott, M. & Campbell, R. E. Ontogeny and reversal of brain circuit abnormalities in a preclinical model of PCOS. *JCI Insight*. **3**, e99405 (2018). 29618656 10.1172/jci.insight.99405 PMC5928858

[27] Esparza, L. A., Schafer, D., Ho, B. S., Thackray, V. G. & Kauffman, A. S. Hyperactive LH pulses and elevated kisspeptin and NKB gene expression in the Arcuate Nucleus of a PCOS Mouse Model. *Endocrinology* **161**, bqaa018 (2020). 32031594 10.1210/endocr/bqaa018 PMC7341557

[28] Naulé, L. et al. Neuroendocrine and behavioral effects of maternal exposure to oral bisphenol A in female mice. *J. Endocrinol.* **220**, 375–388 (2014). 24403293 10.1530/JOE-13-0607

[29] Raskin, K. et al. Conditional inactivation of androgen receptor gene in the nervous system: effects on male behavioral and neuroendocrine responses. *J. Neurosci.* **29**, 4461–4470 (2009). 19357272 10.1523/JNEUROSCI.0296-09.2009 PMC6665718

[30] Adam, N., Brusamonti, L. & Mhaouty-Kodja, S. Exposure of adult female mice to low doses of di(2-ethylhexyl) phthalate alone or in an environmental phthalate mixture: evaluation of Reproductive Behavior and underlying neural mechanisms. *Environ. Health Perspect.* **129**, 17008 (2021). 33502250 10.1289/EHP7662 PMC7839353

[31] Adam, N. et al. Developmental exposure to environmentally relevant doses of phthalates alters the neural control of male and female reproduction in mice. *Environ. Res.* **258**, 119476 (2024). 38909949 10.1016/j.envres.2024.119476

