# Supplementary material for: Effects of minipuberty disruption on the expression of sexual behavior in female mice
Source: Sci Rep. 2024 Dec 28;14:31297. doi: 10.1038/s41598-024-82653-8 (PMC11682410; doi:10.1038/s41598-024-82653-8)
Supplement: Supplementary file 1 — Supplementary Material 1 [file 41598_2024_82653_MOESM1_ESM.pdf]

Effects of mini-puberty disruption on the expression of sexual behavior in female mice

Thomas Torres, Caroline Parmentier, Céline J Guigon, Sakina Mhaouty-Kodja, Lydie Naulé

Figure S1

Figure S2

Figure S3

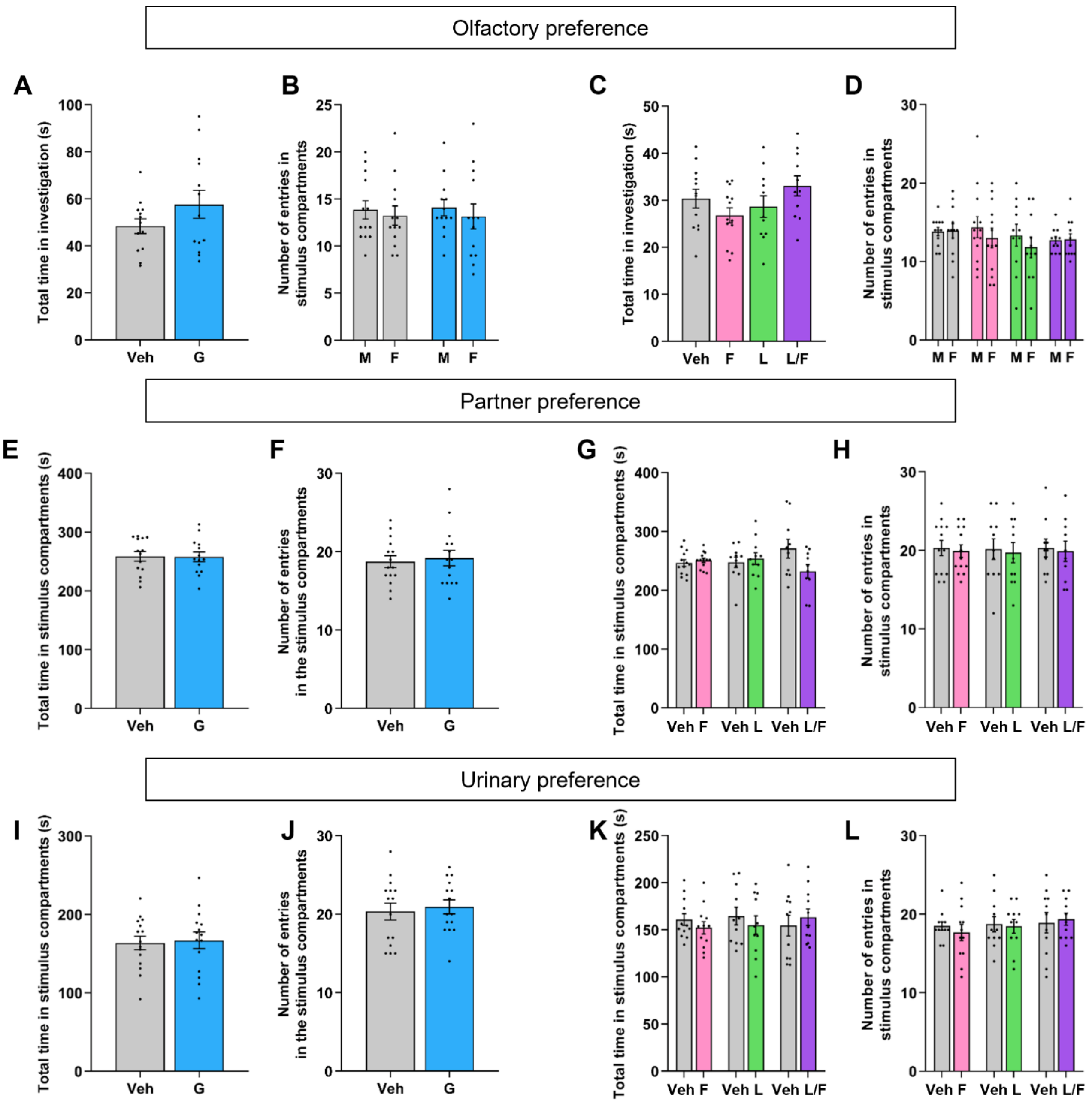

Figure S1. Effect of minipubertal treatment on female sexual behavior. A-D. In the olfactory preference test, female mice treated with vehicle (Veh), Ganirelix (G), flutamide (F), letrozole (L) or flutamide/letrozole (F/L) had choice to chemoinvestigate anesthetized male or female stimuli. The total time in investigation (**A, C**) and the number of entries in both stimulus compartment (**B, D**) were assessed. In the three-chambered tests, sexually experienced males had choice to investigate either Veh- or G-treated females (**E, F**) or either Veh- or F-, Veh- or L-, or Veh- or F/L-treated females (**G, H**). The total time males spent in stimulus compartments (**E, G**) and the number of entries in stimulus

compartments is indicated (**F-H**). In the urinary preference tests, sexually experienced males had choice to investigate urine from either Veh- or G-treated females (**I, J**) or either from Veh- or F-, Veh- or L-, or Veh- or F/L-treated females (**K, L**). The total time males spent in stimulus compartments (**I, K**) and the number of entries in stimulus compartments is indicated (**J-L**). Data are presented as means ± S.E.M. (n = 12-15 per treatment group for Experiment 1; n = 10-13 per treatment group for Experiment 2).

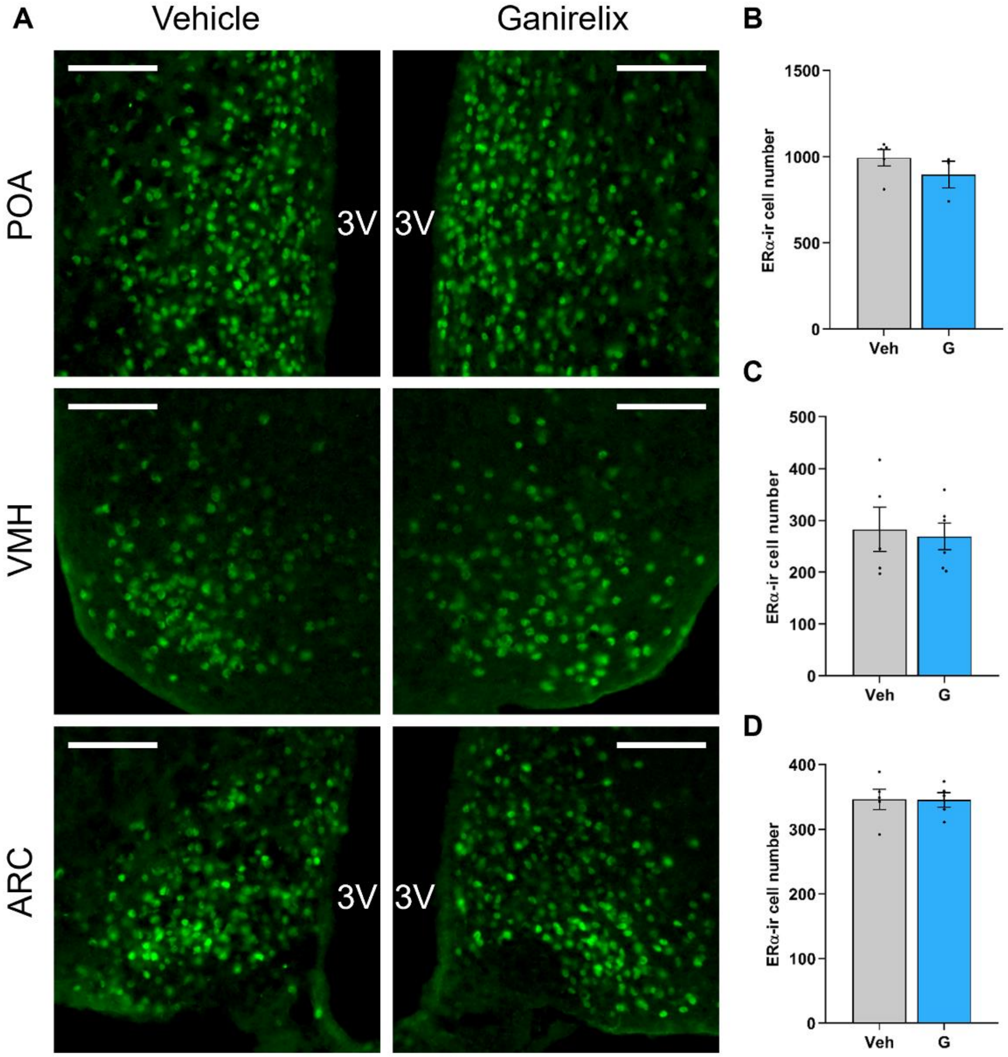

20 **Figure S2. Effect of minipubertal treatment with Ganirelix® on ERα-immunoreactivity in the neural substrates of sexual behavior**. **A**. Representative nuclear ERα-immunolabeling in the POA, VMH and ARC of vehicle- (Veh) and Ganirelix-treated female (G). Scale bar = 100µm. **B-D**. Quantitative analyses of the number of ERα-immunoreactive cells in the POA (**B**), VMH (**C**) and ARC (**D**). Data are presented as means ± S.E.M. (n = 3-6 per treatment).

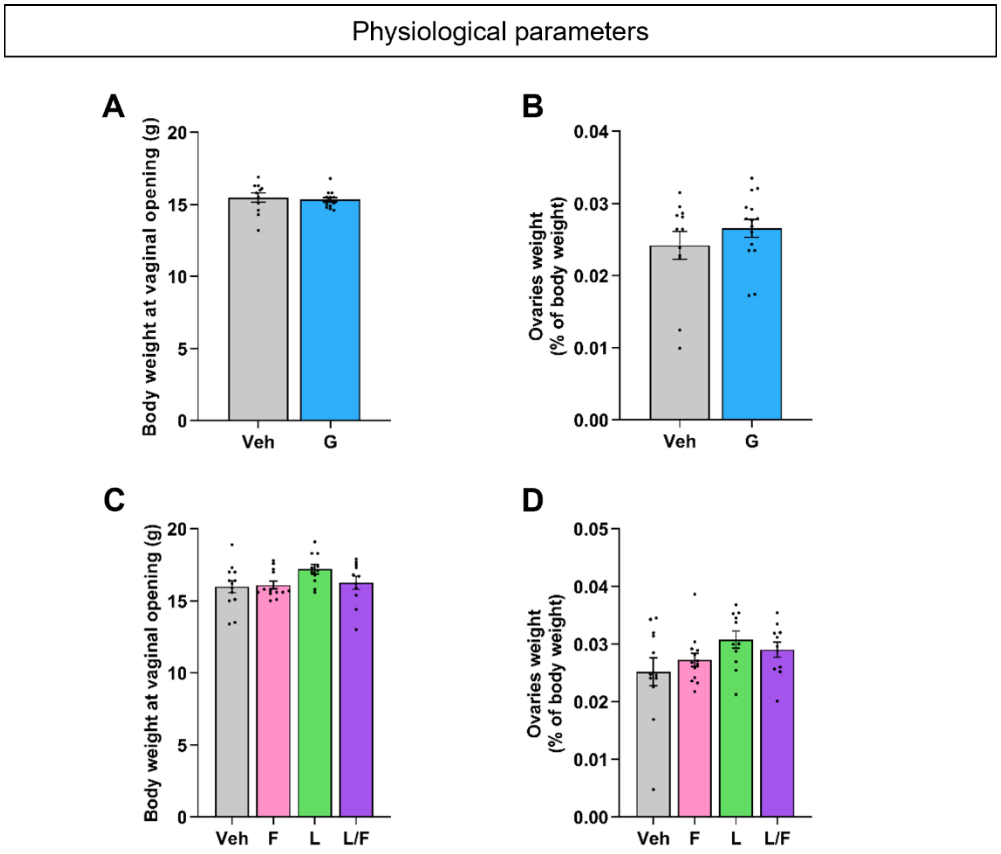

Figure S3. Effect of minipubertal treatment on physiological parameters. A, C. The body weight at vaginal opening is indicated for Experiment 1 (**A**) and Experiment 2 (**C**). **B, D**. 2 weeks before sexual behavior tests, females treated with vehicle (Veh), Ganirelix (G), flutamide (F), letrozole (L) or flutamide/letrozole (F/L) were ovariectomized and the ovaries were weighted for Experiment 1 (**B**) and Experiment 2 (**D**). Data are presented as means ± S.E.M. (n = 12-15 per treatment for Experiment 1; n = 10-13 per treatment for Experiment 2).
